# Backslopping Time, Rinsing of the Grains During Backslopping, and Incubation Temperature Influence the Water Kefir Fermentation Process

**DOI:** 10.3389/fmicb.2022.871550

**Published:** 2022-05-06

**Authors:** David Laureys, Frédéric Leroy, Peter Vandamme, Luc De Vuyst

**Affiliations:** ^1^Research Group of Industrial Microbiology and Food Biotechnology, Faculty of Sciences and Bioengineering Sciences, Vrije Universiteit Brussel, Brussels, Belgium; ^2^Laboratory of Microbiology, Department of Biochemistry and Microbiology, Faculty of Sciences, Ghent University, Ghent, Belgium

**Keywords:** water kefir, yeasts, lactic acid bacteria, bifidobacteria, temperature, backslopping

## Abstract

For eight backslopping steps, eight series of water kefir fermentation processes differing in backslopping time and rinsing of the grains during each backslopping step and eight series of fermentation processes differing in incubation temperature and backslopping time were followed. Short backslopping times resulted in high relative abundances of *Liquorilactobacillus nagelii* and *Saccharomyces cerevisiae*, intermediate backslopping times in high relative abundances of *Leuconostoc pseudomesenteroides*, and long backslopping times in high relative abundances of *Oenococcus sicerae* and *Dekkera bruxellensis*. When the grains were rinsed during each backslopping step, the relative abundances of *Lentilactobacillus hilgardii* and *Leuc*. *pseudomesenteroides* increased and those of *D*. *bruxellensis* and *Liql*. *nagelii* decreased. Furthermore, rinsing of the grains during each backslopping step resulted in a slightly higher water kefir grain growth and lower metabolite concentrations. The relative abundances of *Liquorilactobacillus mali* were highest at 17°C, those of *Leuc*. *pseudomesenteroides* at 21 and 25°C, and those of *Liql*. *nagelii* at 29°C. With a kinetic modeling approach, the impact of the temperature and rinsing of the grains during the backslopping step on the volumetric production rates of the metabolites was determined.

## Introduction

Water kefir is a naturally fermented beverage with a fruity, slightly sweet, alcoholic, and acidic flavor (Laureys and De Vuyst, 2014; Marsh et al., 2014; Lynch et al., 2021). The water kefir fermentation process is started by inoculating a mixture of water, sugar, and (dried) fruits with water kefir grains, followed by anaerobic incubation at room temperature, which usually lasts 2–4 days. At the end of the water kefir fermentation process, the water kefir grains are separated from the liquor by sieving. The liquor is used as a refreshing beverage. Part of the grains is reused to start the next fermentation process. The key microorganisms during water kefir fermentation are the lactic acid bacteria (LAB) *Lacticaseibacillus paracasei, Lentilactobacillus hilgardii*, and *Liquorilactobacillus nagelii*, and the yeast *Saccharomyces cerevisiae*, but other species of LAB, yeasts, bifidobacteria, and/or acetic acid bacteria (AAB) can be present, depending on the fermentation practices (Laureys and De Vuyst, 2017; Lynch et al., 2021).

The interest in water kefir is increasing, as this beverage may offer health benefits to its consumers (Marsh et al., 2014; Corona et al., 2016; Mintel, 2020; Lynch et al., 2021). For example, strains of one of the key microorganisms during water kefir fermentation, *Lacc. paracasei*, may possess probiotic properties (Zagato et al., 2014; Zavala et al., 2016). However, despite this increased interest, the commercial exploitation of water kefir beverages remains limited, partially because the water kefir fermentation process is still difficult to control. To acquire greater control over this process, the impact of the most relevant production parameters needs to be investigated.

The backslopping time may have a pronounced influence on the species diversity during water kefir fermentation, as is also the case during sourdough production through backslopping (Vrancken et al., 2011; De Vuyst et al., 2017). Long backslopping times will increase the acidic stress, which may favor acid-tolerant microorganisms and impact the water kefir grain growth during fermentation (Laureys et al., 2019). In contrast, short backslopping times will reduce the acidic stress and may allow the growth of less acid-tolerant microorganisms, but may flush out slow-growing ones. The latter may be even more pronounced when the grains are rinsed during each backslopping step, as is often the case during water kefir fermentation processes (Lynch et al., 2021). Rinsing of the water kefir grains may remove residual substrates and metabolites, and hence produce extra waste and even detach microorganisms from the grains. The former may result in the lower substrate and metabolite concentrations, lower acidic stress, and thus higher water kefir grain growth, whereas the latter may result in a slower water kefir fermentation process (Laureys and De Vuyst, 2017). From an industrial point of view, rinsing of the water kefir grains during each backslopping step is thus not desirable. However, rinsing of the water kefir grains during each backslopping step may favor only those microorganisms that are strongly attached to the grains, while removing contaminants. Rinsing of the grains during each backslopping step may thus be necessary to maintain a stable water kefir microbiota, but this has not been investigated yet.

The incubation temperature likely exerts a large influence on the water kefir fermentation rate, as is also the case during milk kefir fermentation (Zajšek and Goršek, 2010). A high incubation temperature will increase the fermentation rate, which is desirable from an industrial point of view. However, the incubation temperature may also affect the microbial species diversity and community dynamics during water kefir fermentation, as is the case during sourdough production (Vrancken et al., 2011; Bessmeltseva et al., 2014; De Vuyst et al., 2017). Such a shift in the microbial communities may be reflected in metabolite production. Additionally, the incubation temperature may directly affect the metabolism of certain microorganisms, as is the case for the production of lactic acid and acetic acid by *Lacc. casei* (Qin et al., 2012), and the production of ethanol and glycerol by *S*. *cerevisiae* (Yalcin and Ozbas, 2008). Furthermore, high temperatures might stimulate the production of dextran, the exopolysaccharide composing the water kefir grains, as the optimal temperature of dextran sucrase from *Lenl. hilgardii* is between 40 and 45°C (Waldherr et al., 2010). However, the influence of the temperature during water kefir fermentation has not been investigated yet.

This study aimed to determine the impact of the backslopping time, rinsing off the water kefir grains during each backslopping step, and the incubation temperature on the microbial species diversity, substrate consumption, and metabolite production during the water kefir fermentation process. A modeling approach was used to allow a quantitative analysis of the effects of rinsing and temperature on the process characteristics.

## Materials and Methods

### Water Kefir Grain Inoculum and Prefermentations

Two inocula of water kefir grains were obtained from a household water kefir fermentation process, as described before (Laureys and De Vuyst, 2014). To obtain the necessary amount of water kefir grains for the actual fermentation experiments, each inoculum was cultivated through a series of consecutive prefermentations through backslopping until >1,300 g of water kefir grain wet mass was produced. The prefermentations were performed in glass bottles (1, 2, and 5 L), equipped with a polytetrafluoroethylene (PTFE) water lock. They were started with a medium consisting of 10 g of sugar (Candico Bio, Merksem, Belgium), 5 g of dried figs (King Brand, Naziili, Turkey), and 160 ml of tap water (Brussels, Belgium) per 50 g of water kefir grain wet mass. The bottles were incubated in a water bath at 21°C. A backslopping practice was applied every 3 days, whereby the water kefir grains were separated from the water kefir liquor by sieving, after which the water kefir grains were recultivated in a fresh medium under the same conditions as mentioned above to be used as final inoculum.

### Fermentations

**Figure 1:**
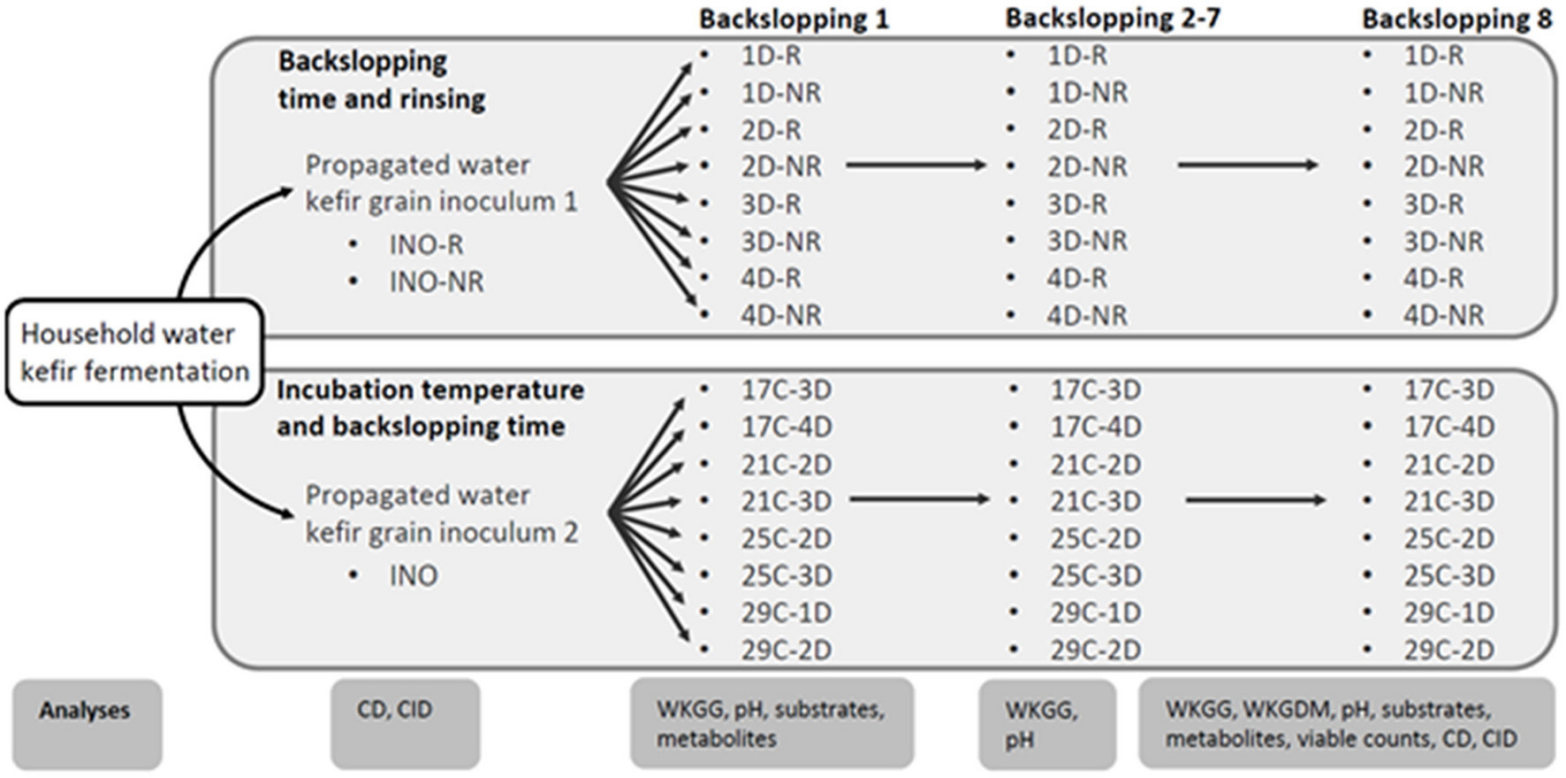
Schematic overview of the household water kefir fermentation processes, the water kefir grain inoculum 1 (INO-R and INO-NR) and 2 (INO) obtained through a series of prefermentations, the eight series of water kefir fermentations differing in backslopping time and rinsing or not rinsing off the water kefir grains during each backslopping step, and the eight series of water kefir fermentations differing in incubation temperature and backslopping time, as well as the different analyses performed throughout the experimental process. The varied process parameters were the backslapping time D (1, 2, 3, or 4 days), the incubation temperature C (17, 21, 25, or 29°C), and the rinsing of the water kefir grains during each backsloppings step [rinsed (R) or non-rinsed (NR)]. Analyses included culture-dependent (CD) and culture-independent (CID) microbial species diversity, substrate consumption (substrates), metabolite production (metabolites), water kefir grain growth (WKGG), water kefir grain dry mass (WKGDM), pH, and/or viable counts.

The first inoculum of water kefir grains, obtained through the series of prefermentations mentioned above, was used to start eight series of water kefir fermentation processes differing in backslopping time and rinsing of the water kefir grains during each backslopping step (Figure 1). The backslopping times were 1 day (indicated as D) (further referred to as fermentation series 1D-R and 1D-NR), 2 days (2D-R and 2D-NR), 3 days (3D-R and 3D-NR), and 4 days (4D-R and 4D-NR). For each backslopping time, one fermentation series was started with a rinsed grain inoculum, after which the water kefir grains were rinsed (R) during each backslopping step (1D-R, 2D-R, 3D-R, and 4D-R), and another fermentation series was started with a non-rinsed (NR) grain inoculum, after which the water kefir grains were not rinsed upon backslopping (1D-NR, 2D-NR, 3D-NR, and 4D-NR). Rinsing of the grains was performed with 2 L of tap water per 50 g of water kefir grains.

The second inoculum of water kefir grains, also obtained through a series of prefermentations as mentioned above, was used to start eight series of water kefir fermentation processes differing in incubation temperature and backslopping time (Figure 1). The incubation temperatures (C) were 17°C (fermentation series 17C-3D and 17C-4D), 21°C (21C-2D and 21C-3D), 25°C (25C-2D and 25C-3D), and 29°C (29C-1D and 29C-2D). The backslopping times were 1 day (29C-1D), 2 days (21C-2D, 25C-2D, and 29C-2D), 3 days (17C-3D, 21C-3D, and 25C-3D), and 4 days (17C-4D). All those water kefir fermentation series were started with a rinsed grain inoculum, and the water kefir grains were always rinsed during each backslopping step.

All fermentation series were performed in independent biological triplicates, and each fermentation was carried out in 250-ml glass bottles equipped with a water lock (PTFE). All fermentation processes were started with a medium consisting of 10 g of sugar (Candico Bio), 5 g of dried figs (King Brand), 160 ml of tap water (Brussels, Belgium), and 50 g of a rinsed or non-rinsed grain inoculum (depending on the fermentation series). The bottles were incubated in a water bath at 21°C, unless stated otherwise, depending on the fermentation series. The contents of the bottles were mixed by gently turning them at the start and the end of each backslopping step. For the backslopping practice, the water kefir grains were separated from the water kefir liquors by sieving and rinsed or not rinsed (depending on the fermentation series), after which 50 g of water kefir grains were re-cultivated in fresh medium and under the same conditions as before. This practice was continued for eight backslopping steps.

### Analyses

#### pH, Water Kefir Grain Wet and Dry Mass, and Water Kefir Grain Growth Determinations

The pH, the water kefir grain wet mass, and the water kefir grain growth were determined at the end of each backslopping step. The water kefir grain dry mass was determined at the end of backslopping step 8. All determinations were done as described before (Laureys et al., 2019).

#### Microbial Enumerations

The viable counts of the presumptive yeasts, LAB, and AAB were determined for the rinsed and non-rinsed water kefir grains of the first inoculum of water kefir grains, the non-rinsed water kefir grains of the second inoculum of water kefir grains, and the non-rinsed water kefir grains of all fermentation series at the end of backslopping step 8.

The viable counts of the presumptive yeasts were determined on yeast extract-peptone-dextrose (YPD) agar medium, those of the presumptive LAB on de Man-Rogosa-Sharpe (MRS) agar medium, and those of the presumptive AAB on modified deoxycholate-mannitol-sorbitol (mDMS) agar medium, as described before (Laureys et al., 2019).

#### Culture-Dependent Microbial Species Diversity Analyses

The culture-dependent microbial species diversities of the LAB and yeasts were determined for the rinsed and non-rinsed water kefir grains of the first inoculum of water kefir grains, the non-rinsed water kefir grains of the second inoculum of water kefir grains, and the non-rinsed water kefir grains of all fermentation series at the end of backslopping step 8.

The culture-dependent microbial species diversities in the water kefir liquors and on the water kefir grains were determined by randomly picking 10–20% of the total number of colonies from the respective agar media harboring 30–300 colonies. The isolates were sub-cultivated on their respective agar media until the third generation, which was used for dereplication *via* matrix-assisted laser desorption/ionization time-of-flight mass spectrometry (MALDI-TOF MS) fingerprinting, as described before (Spitaels et al., 2014). The fingerprint peptide patterns obtained were clustered numerically by means of the BioNumerics software version 5.10 (Applied Maths, Sint-Martens-Latem, Belgium). Representative bacterial isolates within each cluster were identified by sequencing part of their 16S rRNA gene from genomic DNA, and representative yeast isolates within each cluster were identified by sequencing their 26S large subunit (LSU) rRNA gene and internal transcribed spacer (ITS) region from genomic DNA, as described before (Laureys et al., 2019).

#### Exopolysaccharide Production

All LAB isolates were grown on an MRS agar medium supplemented with 10 g L^−1^ of sucrose at 30°C for 7 days to visually assess their exopolysaccharide (EPS) production capacity.

#### Culture-Independent Microbial Species Diversity Analyses

The culture-independent microbial species diversities of yeasts and bacteria were determined for the water kefir liquors and the rinsed and non-rinsed water kefir grains of the first inoculum of water kefir grains, the water kefir liquors and the non-rinsed water kefir grains of the second inoculum of water kefir grains, and the water kefir liquors and non-rinsed water kefir grains of all fermentation series at the end of backslopping step 8.

The culture-independent microbial species diversities in the water kefir liquors and on the water kefir grains was determined after preparing total DNA extracts from the cell pellets of the water kefir liquors and 0.2 g of crushed water kefir grains, respectively, as described before (Laureys et al., 2019). The culture-independent microbial community profiles were obtained by amplifying selected genomic fragments in the total DNA with the universal prokaryotic primer pair (V3), the LAB-specific primer pair (LAC), the *Bifidobacterium*-specific primer pair (Bif), and the universal eukaryotic primer pair (Yeast), and separating the PCR amplicons through denaturing gradient gel electrophoresis (DGGE). Selected bands of the community profiles were cut from the gels and identities were assigned through sequencing, as described before (Laureys and De Vuyst, 2014).

#### Substrate and Metabolite Concentration Determinations

The substrate and metabolite concentrations in the liquors of all fermentation series were determined at the end of backslopping steps 1 and 8.

Samples for substrate and metabolite concentration analyses were prepared as described before (Laureys et al., 2019). The concentrations of sucrose, glucose, fructose, glycerol, and mannitol were determined through high-performance anion-exchange chromatography with pulsed amperometric detection (HPAEC-PAD), those of D- and L-lactic acid and acetic acid through high-performance liquid chromatography with ultraviolet detection (HPLC-UV), those of ethanol through gas chromatography with flame ionization detection (GC-FID), and those of the aroma compounds through static headspace gas chromatography with mass spectrometry detection (SH-GC-MS).

### Statistics

All results are presented as the mean ± standard deviation of the three independent biological replicates performed for each fermentation series.

An ANOVA was performed to test for differences between the eight fermentation series, followed by a series of *post-hoc* pairwise comparisons with Fisher's least significant difference (LSD) test, as described before (Laureys et al., 2019). All statistical tests were performed in R 3.2.0 (R Core Team, 2015) with a significance level of 0.05.

### Kinetic Model Development

#### Model Equations for Microbial Metabolite Production

The concentrations of the microbial metabolites ethanol [Eth], lactic acid [LA], acetic acid [AA], glycerol [Gly], and mannitol [Mtl] (g L^−1^) during water kefir fermentation were described as a function of time, based on their initial concentrations [Eth]_0_, [LA]_0_, [AA]_0_, [Gly]_0_, and [Mtl]_0_ (g L^−1^) and their volumetric production rates k_Eth_, k_LA_, k_AA_, k_Gly_, and k_Mtl_ (g L^−1^ h^−1^), as described before (Laureys et al., 2021). A general expression for each metabolite (P), taking its initial concentration ([P]_0_) into account, was used:


(1) $$\begin{array}{l} \left[\text{P}\right]={\left[\text{P}\right]}_{0}+\text{k t} \end{array}$$

#### Influence of Rinsing of the Water Kefir Grains on the Volumetric Production Rates and the Initial Concentrations of the Microbial Metabolites

To compare the volumetric production rates between the water kefir fermentation processes that started with rinsed or non-rinsed grains, a linear model was developed, whereby the initial metabolite concentrations ([P]_0_) and volumetric production rates (Time) depended on the rinsing of the grains (Rinsing and Time: Rinsing, respectively), as follows:


(2) $$\begin{array}{l} \text{P }\sim \text{ Rinsing}+\text{Time}+\text{Time}:\text{Rinsing} \end{array}$$

For the metabolites of which the volumetric production rates were not significantly different between the water kefir fermentation processes that started with rinsed or non-rinsed grains (see the “Results” section), the interaction term could be removed:


(3) $$\begin{array}{l} \text{P }\sim \text{ Rinsing}+\text{Time} \end{array}$$

For the metabolites of which the estimated initial concentrations were not significantly different between the water kefir fermentation processes that started with rinsed or non-rinsed grains (see the “Results” section), the linear model could be further simplified, as follows:


(4) $$\begin{array}{l} \text{P }\sim \text{ Time} \end{array}$$

#### Influence of the Incubation Temperature on the Volumetric Production Rates of the Microbial Metabolites

The volumetric production rates were assumed to be dependent on the temperature as described by the Arrhenius equation, wherein A is a pre-exponential factor (g L^−1^ h^−1^), E_a_ is the activation energy for the reaction (J mol^−1^), R is the universal gas constant (J mol^−1^ K^−1^), and T is the incubation temperature (K):


(5) $$\begin{array}{l} \text{k}=\text{A}{\text{e}}^{-\text{Ea}/\left(\text{RT}\right)} \end{array}$$

The metabolite concentration [P] as a function of time, as described above (Equation 1), was extended with the Arrhenius equation to account for the incubation temperature.

To estimate the A and E_a_ values, a non-linear model was developed:


(6) $$\begin{array}{l} {\text{P }\sim \;{\left[\text{P}\right]}_{0}+\text{Ae}}^{-\text{Ea}/\left(\text{RT}\right)}*\text{Time} \end{array}$$

The calculation of the temperature coefficient or Q_10_ values was based on the E_a_ values.

#### Fitting of the Model Equations to the Experimental Data

The estimations of the biokinetic parameters were performed in R 3.2.0 (R Core Team, 2015), and the results are presented as the mean ± standard error.

The initial concentrations and volumetric production rates for the production kinetics of the metabolites during the water kefir fermentation processes that started with rinsed (fermentation series 1D-R, 2D-R, and 3D-R) or non-rinsed grains (1D-NR, 2D-NR, and 3D-NR) were estimated by fitting the linear models to the linear portions of the experimental data (which was from 0 to 72 h of fermentation) at the end of backslopping step 1.

The parameters of the Arrhenius equations used to describe the influence of the temperature on the volumetric production rates were estimated by fitting non-linear models to the experimental data (Klicka and Kubácek, 1997). The experimental data used for this estimation were those at the end of backslopping step 1 of the water kefir fermentation processes with different backslopping times performed for each fermentation temperature. The initial concentrations of the metabolites were assumed to be similar to the estimated initial concentrations of the water kefir fermentation process that started with rinsed water kefir grains.

## Results

### Water Kefir Grain Wet and Dry Mass and pH

For the eight fermentation series differing in backslopping time and rinsing of the grains during each backslopping step, the water kefir grain growth was similar for all backslopping times, although slightly higher when the grains were rinsed during each backslopping step (Supplementary Figure 1 and Supplementary Tables 1, 2). This indicated that most of the water kefir grain wet mass was produced during the first 24 h of fermentation. Long backslopping times resulted in lower pH values than short backslopping times, and rinsing of the water kefir grains resulted in lower pH values than when the water kefir grains were not rinsed (Supplementary Figure 1 and Supplementary Tables 1, 2).

For the eight fermentation series differing in incubation temperature and backslopping time, the water kefir grain growth was always similar (Supplementary Figure 1 and Supplementary Tables 3, 4). Furthermore, the pH at the end of each backslopping step was low when the backslopping time was long.

The water kefir grain dry mass was always ~13–17% (m m^−1^) and was high when the residual total carbohydrate concentrations were high (Supplementary Tables 2, 4).

### Microbial Enumerations

The viable counts of the yeasts on the rinsed and non-rinsed water kefir grains of the first inoculum of water kefir grains and on the non-rinsed water kefir grains of the second inoculum of water kefir grains were 7.6 ± 0.1, 7.7 ± 0.1, and 7.4 ± 0.1 log (cfu g^−1^) of grains, respectively. Those of the LAB were 8.8 ± 0.1, 8.9 ± 0.1, and 8.4 ± 0.4 log (cfu g^−1^) of grains, respectively. Rinsing of the grains did not significantly affect their viable counts of yeasts and LAB. The viable counts of the AAB were below the limit of quantification for all water kefir grain inocula.

The viable counts of the yeasts on the water kefir grains at the end of backslopping step 8 were ~7.5 log (cfu g^−1^) of grains for all fermentation series (Supplementary Tables 2, 4). Those of the LAB were around 8.5 log (cfu g^−1^) of grains for all fermentation series (Supplementary Tables 2, 4). This resulted in relatively similar ratios of the viable counts of the LAB to those of the yeasts of ~10. The viable counts of the AAB on the water kefir grains were ~4.5 log (cfu g^−1^) of grains for most water kefir fermentation series, but were significantly lower (*p* <0.05) for the fermentation series 1D-NR, and even much lower for the fermentation series 1D-R.

### Culture-Dependent Microbial Species Diversity

#### Water Kefir Grain Inocula

**Figure 2:**
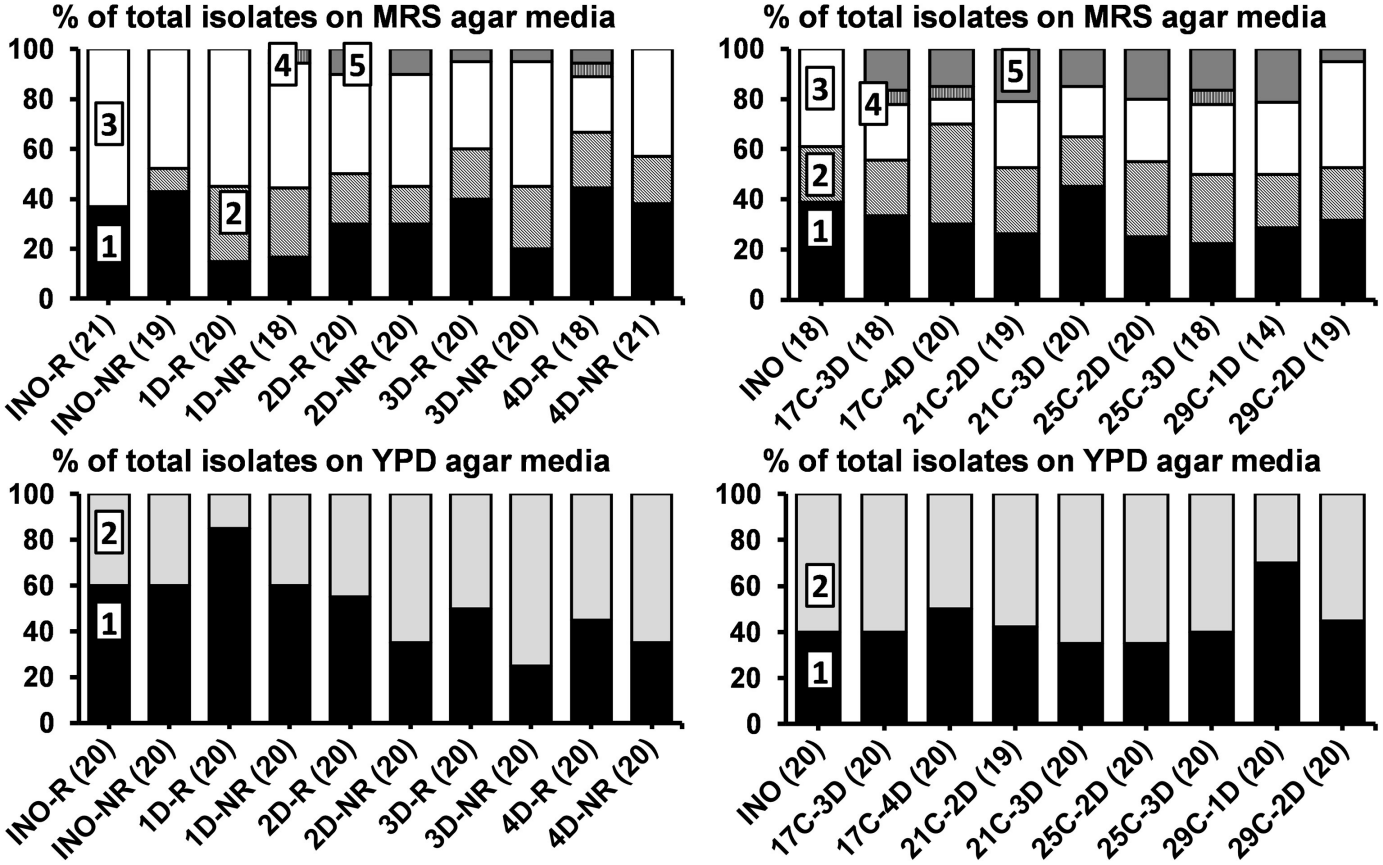
Culture-dependent microbial species diversity of the rinsed (INO-R) and non-rinsed (INO-NR) grain inocula and of the non-rinsed grains of the eight series of water kefir fermentations (21°C) differing in backslopping time and rinsing of the grains during each backslopping step, at the end of backslopping step 8 (left), as well as of the non-rinsed grain inoculum (INO) and the non-rinsed grains of the eight series of water kefir fermentations differing in incubation temperature and backslopping time, at the end of backslopping step 8 (right). The number of isolates is indicated between brackets. Isolates from MRS agar media: (1) *Lacticaseibacillus paracasei* (100% identity; GenBank accession no. AP012541); (2) *Lentilactobacillus hilgardii* (100% identity; accession no. LC064898); (3) *Liquorilactobacillus nagelii* (99% identity; accession no. NR112754); (4) *Liquorilactobacillus mali* (99% identity; accession no. NR112691); and (5) *Leuconostoc pseudomesenteroides* (99% identity; accession no. LC096220). Isolates from YPD agar media: (1) *Saccharomyces cerevisiae* [LSU (99% identity; accession no. CP011558) and ITS (99% identity; accession no. KC515374)]; and (2) *Dekkera bruxellensis* [LSU (99% identity; accession no. GU291284) and ITS (99% identity; accession no. FJ545249)]. LSU, large subunit rRNA gene; ITS, internal transcribed spacer. C, temperature (17, 21, 25, or 29°C); D, days of backslopping (1, 2, 3, or 4); R, rinsed; NR, non-rinsed.

The main yeasts and LAB found in the grain inocula culture-dependently were *S. cerevisiae, Dekkera bruxellensis, Lacc. paracasei, Lenl. hilgardii*, and *Liql. nagelii*. The communities of the yeasts and LAB on the rinsed and non-rinsed water kefir grains of the first inoculum of water kefir grains and the non-rinsed grains of the second inoculum of water kefir grains were similar (Figure 2). These microorganisms were also found on the grains of all fermentation series at the end of backslopping step 8.

#### Backslopping Time and Rinsing of the Water Kefir Grains

For the eight fermentation series differing in backslopping time and rinsing of the water kefir grains during each backslopping step, the relative abundances of *Lacc*. *paracasei* and *D*. *bruxellensis* on the grains increased and those of *Liql*. *nagelii* and *S*. *cerevisiae* decreased with longer backslopping times (Figure 2). Furthermore, the relative abundances of *D*. *bruxellensis* were higher when the water kefir grains were not rinsed during each backslopping step. Additionally, *Leuconostoc pseudomesenteroides* was found on the grains of the series with a backslopping time of 2 or 3 days and in the series with a backslopping time of 4 days, whereby the grains were rinsed during each backslopping step. *Liquorilactobacillus mali* was found on the grains of the fermentation series with a backslopping time of 1 day without rinsing of the grains during each backslopping step and in the series with a backslopping time of 4 days with rinsing of the grains.

All *Liql*. *mali* and *Leuc*. *pseudomesenteroides* isolates and 40% of the *Lenl*. *hilgardii* isolates produced EPS, whereby the proportion of EPS-producing *Lenl*. *hilgardii* isolates was similar for the eight fermentation series. Additionally, 25 and 44% of the *Liql*. *nagelii* isolates from fermentation series 4D-R and 4D-NR, respectively, produced EPS.

#### Incubation Temperature and Backslopping Time

For the eight fermentation series differing in incubation temperature and backslopping time, the relative abundances of *Liql*. *nagelii* on the grains increased as the temperature increased. The relative abundances of *D*. *bruxellensis* were low on the grains of the fermentation series with an incubation temperature of 29°C and a backslopping time of 1 day (Figure 2). Additionally, *Leuc*. *pseudomesenteroides* was found on the grains of the eight fermentation series investigating the influence of the incubation temperature and backslopping time. *Liql. mali* was found on the grains of the fermentation series with an incubation temperature of 17°C and a backslopping time of 3 or 4 days and the fermentation series with an incubation temperature of 25°C and a backslopping time of 3 days.

All *Liql*. *mali* and *Leuc*. *pseudomesenteroides* isolates and 51% of the *Lenl*. *hilgardii* isolates produced EPS, whereby the proportion of EPS-producing *Lenl. hilgardii* isolates was similar for the eight fermentation series. Additionally, 20% of the *Liql*. *nagelii* isolates from the fermentation series 25C-3D produced EPS.

Isolates of *Liql*. *mali* and *Liql*. *nagelii* produced EPS that remained localized around the colonies, whereas isolates of *Lenl*. *hilgardii* and *Leuc*. *pseudomesenteroides* produced EPS that spread over the whole plate. This might indicate the production of cell-bound or soluble glucansucrases, respectively (CôtéCôté et al., 2013 ).

### Culture-Independent Microbial Species Diversity

At the end of backslopping step 8, the rRNA-PCR-DGGE community profiles of the grains and in the liquors obtained with the four different primer pairs (V3, LAC, Bif, and Yeast) were similar for the three independent biological replicates performed for each fermentation series (results not shown).

#### Water Kefir Grain Inocula

**Figure 3:**
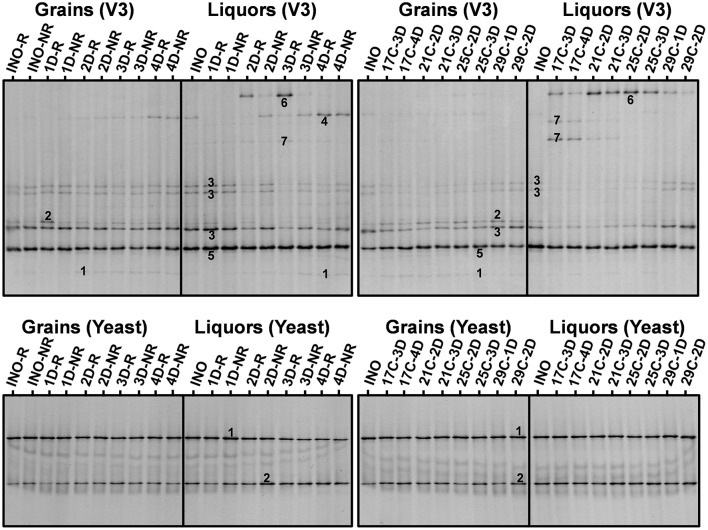
Culture-independent species diversity of the rinsed (INO-R) and non-rinsed (INO-NR) grains and liquor of the grain inoculum (INO) and of the non-rinsed grains and liquors of the eight series of water kefir fermentations differing in backslopping time and rinsing of the grains during each backslopping step, at the end of backslopping step 8 (left), as well as of the non-rinsed grains (INO) and liquor of the grain inoculum (INO), and the non-rinsed grains and liquors of the eight series of water kefir fermentations differing in incubation temperature and backslopping time, at the end of backslopping step 8 (right). Community profiles obtained with the V3 primer pair: (1) *Lacticaseibacillus casei*/*paracasei/zeae/rhamnosus* (99% identity for all species; GenBank accession nos. LC064894/AB289229/ AB289313/JQ580982); (2) *Lentilactobacillus hilgardii/diolivorans* (100% identity; accession nos. LC064898/NR037004); (3) *Liquorilactobacillus nagelii/ghanensis* (99% identity; accession nos. NR119275/NR043896); (4) *Oenococcus sicerae* (99% identity; accession no. CP029684); (5) *Bifidobacterium aquikefiri* (100% identity; accession no. LN849254); (6) *Leuconostoc pseudomesenteroides* (99% identity; accession no. LC096220); and (7) *Liquorilactobacillus mali/hordei* (100% identity; accession nos. LC064888/NR044394). Community profiles obtained with the yeast primer pair: (1) *Saccharomyces cerevisiae* (100% identity; accession no. NG042623); and (2) *Dekkera bruxellensis* (100% identity; accession no. AY969049). C, temperature (17, 21, 25, and 29°C); D, days of backslopping (1, 2, 3, or 4); R, rinsed; NR, non-rinsed.

The main bands in the community profiles obtained with the four primer pairs for the grains and liquors of the inocula were attributed to *S*. *cerevisiae, D*. *bruxellensis, Lacc*. *paracasei, Lenl*. *hilgardii, Liql*. *nagelii*, and *Bifidobacterium aquikefiri* (Figure 3). In all replicates, the relative intensities of the bands attributed to *Lenl*. *hilgardii* were consistently slightly higher and those attributed to *D*. *bruxellensis* and *Lacc*. *paracasei* were slightly lower when the grain inoculum was rinsed (INO-R) than when it was not rinsed (INO-NR). Furthermore, most microorganisms found in the water kefir grain inocula and the liquors thereof were also found in most fermentation series at the end of backslopping step 8.

#### Backslopping Time and Rinsing of the Water Kefir Grains

For the eight series of fermentations differing in backslopping time and rinsing during each backslopping step, the relative intensities of the bands attributed to *S*. *cerevisiae, Liql*. *nagelii*, and *Lenl*. *hilgardii* decreased and those of the bands attributed to *D*. *bruxellensis, Lacc*. *paracasei*, and *Oenococcus sicerae* increased when the backslopping time increased (Figure 3). Additionally, high relative intensities of the bands attributed to *Leuc*. *pseudomesenteroides* were found in the fermentation series with backslopping times of 2 or 3 days. Overall, when the water kefir grains were rinsed during each backslopping step, the relative intensities of the bands attributed to *D. bruxellensis* and *Liql. nagelii* were lower and those of the bands attributed to *Lenl. hilgardii* and *Leuc. pseudomesenteroides* were higher than when the grains were not rinsed during each backslopping step. These effects were mainly seen for the water kefir liquors and only to a lesser extent for the water kefir grains.

#### Incubation Temperature and Backslopping Time

For the eight series of fermentations differing in incubation temperature and backslopping time, the relative intensities of the bands attributed to *Liql*. *mali* decreased and those of the bands attributed to *Liql*. *nagelii* increased when the incubation temperature increased (Figure 3). The relative intensities of the bands attributed to *Leuc*. *pseudomesenteroides* were highest when the incubation temperature was 21 or 25°C. Overall, for each incubation temperature, the relative intensities of the bands attributed to *Leuc. pseudomesenteroides* and *Lenl. hilgardii* were lowest and those of the bands attributed to *D. bruxellensis* and *Lacc. paracasei* were the highest in the series with the longest backslopping time. These effects were mainly seen for the water kefir liquors and only to a lesser extent for the water kefir grains.

Taken together, the relative intensities of the bands attributed to *D*. *bruxellensis, Leuc*. *pseudomesenteroides, Liql*. *mali*, and *O. sicerae* were higher for the liquors, whereas those of the bands attributed to *Lenl*. *hilgardii* were higher for the grains (Figure 3).

### Substrate and Microbial Metabolite Concentration Dynamics

The concentrations of the microbial metabolites ethanol, glycerol, lactic acid, acetic acid, and aroma compounds in the water kefir liquors were higher when the backslopping time was longer and when the water kefir grains were not rinsed during each backslopping step. In contrast, the concentrations of mannitol were higher when the grains were rinsed during each backslopping step. Overall, the ratios of the different metabolites were not substantially impacted by the backslopping time, rinsing off the water kefir grains during each backslopping step, or the incubation temperature (Supplementary Tables 1–4).

### Kinetic Models for the Production of Microbial Metabolites

#### Influence of Rinsing of the Water Kefir Grains on the Volumetric Production Rates and the Initial Concentrations of the Microbial Metabolites

**Figure 4:**
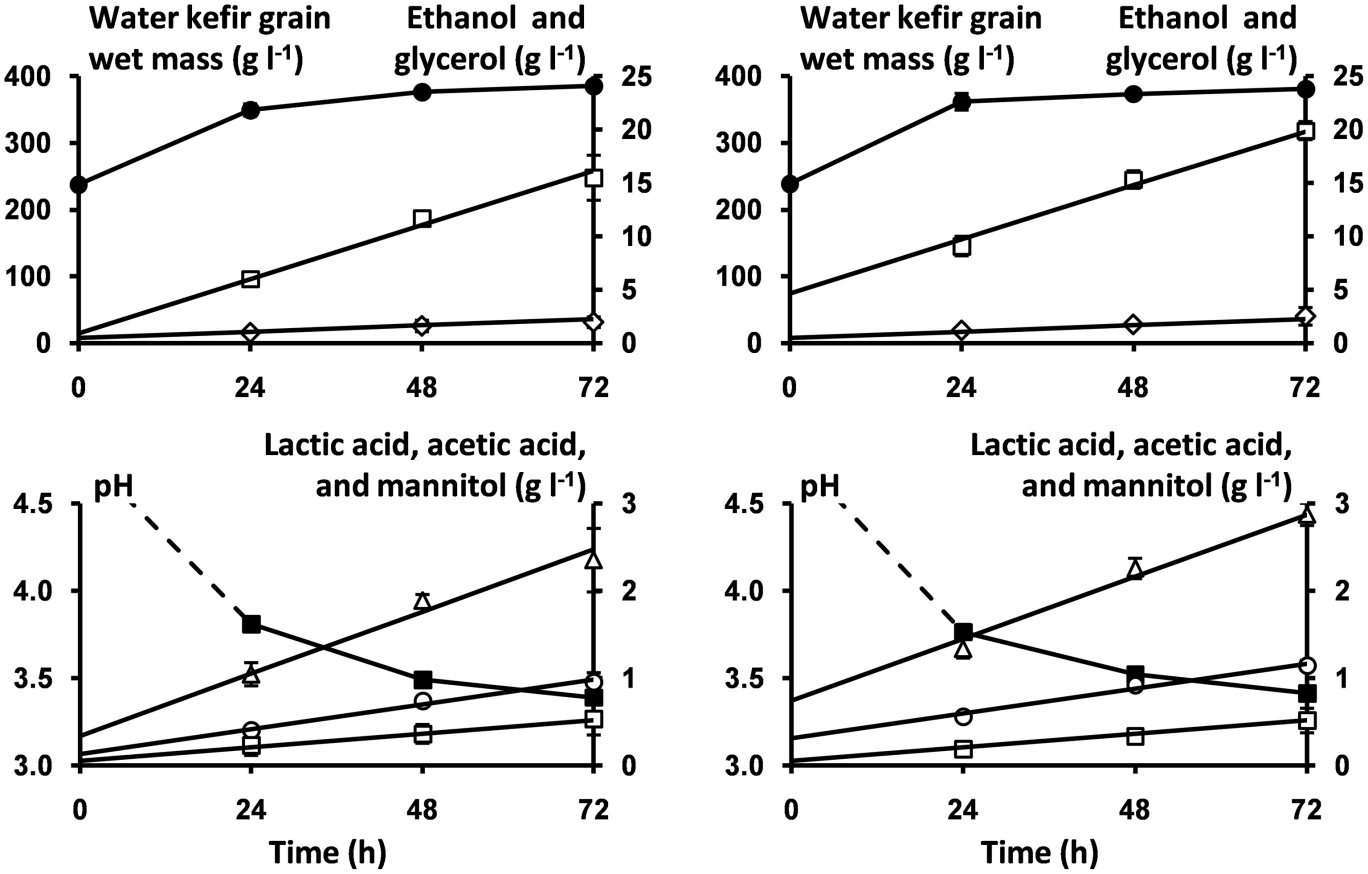
Course of pH (■) and concentrations of water kefir grain wet mass (●), ethanol (□), glycerol (♢), lactic acid (Δ), acetic acid (○), and mannitol (□) as a function of time, as well as the model lines (solid lines) describing the concentrations of ethanol, glycerol, lactic acid, acetic acid, and mannitol during the first 72 h of fermentation of the water kefir fermentation series that started with rinsed (left) or non-rinsed (right) grains.

The estimated volumetric production rates for each microbial metabolite in the water kefir liquors were not significantly different between the water kefir fermentation processes that started with rinsed or non-rinsed grains (Table 1). This allowed us to remove the interaction term from Equation (2) of the linear model for all metabolites (Equation 3). The estimated initial concentrations of ethanol, lactic acid, and acetic acid were significantly different between the water kefir fermentation processes that started with rinsed or non-rinsed grains. However, the estimated initial concentrations of glycerol and mannitol were not significantly different between the water kefir fermentation processes that started with rinsed or non-rinsed grains, and for these metabolites, the linear model was further simplified (Equation 4). Overall, rinsing of the water kefir grains reduced the initial concentrations of the metabolites but not the volumetric production rates for the production of these metabolites (Figure 4).

**Table 1:** Estimated initial concentrations and volumetric production rates for the production kinetics of ethanol ([Eth]_0_ and k_Eth_), lactic acid ([LA]_0_ and k_LA_), acetic acid ([AA]_0_ and k_AA_), glycerol ([Gly]_0_ and k_Gly_), and mannitol ([Mtl]_0_ and k_Mtl_) during water kefir fermentation processes started with rinsed or non-rinsed grains; as well as the *p*-values for the differences between the estimated values for these biokinetic parameters.

**Parameter**	***P***	**Rinsed grain inoculum**	**Non-rinsed grain inoculum**
[Eth]_0_ (g l^−1^)	<0.001	0.92 ± 0.52	4.61 ± 0.73
[LA]_0_ (g l^−1^)	<0.001	0.34 ± 0.09	0.74 ± 0.13
[AA]_0_ (g l^−1^)	<0.001	0.13 ± 0.03	0.31 ± 0.04
[Gly]_0_ (g l^−1^)	0.164	0.48 ± 0.27	0.48 ± 0.27
[Mtl]_0_ (g l^−1^)	0.609	0.05 ± 0.06	0.05 ± 0.06
k_Eth_ (mg l^−1^ h^−1^)	0.309	211 ± 13	211 ± 13
k_LA_ (mg l^−1^ h^−1^)	0.315	29.6 ± 2.3	29.6 ± 2.3
k_AA_ (mg l^−1^ h^−1^)	0.609	11.9 ± 0.7	11.9 ± 0.7
k_Gly_ (mg l^−1^ h^−1^)	0.501	24.2 ± 5.1	24.2 ± 5.1
k_Mtl_ (mg l^−1^ h^−1^)	0.892	6.5 ± 1.2	6.5 ± 1.2

*The results are presented as the mean ± standard error*.

#### Influence of the Incubation Temperature on the Volumetric Production Rates of the Microbial Metabolites

**Figure 5:**
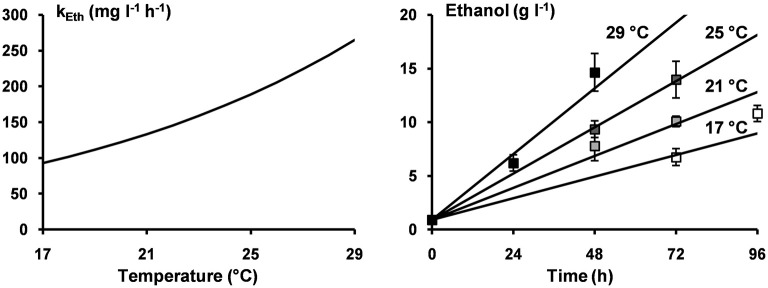
Arrhenius equation describing the volumetric production rates for the production of ethanol (k_Eth_) as a function of the incubation temperature (left); the concentrations of ethanol after 72 and 96 h of incubation at 17°C (□), after 48 and 72 h of incubation at 21°C (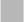), after 48 and 72 h of incubation at 25°C (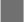), and after 24 and 48 h of incubation at 29°C (■) (right); and the model lines (solid lines) describing the concentrations of ethanol at incubation temperatures of 17, 21, 25, and 29°C (right).

For each microbial metabolite, the values of A and E_a_ were estimated, and the estimated E_a_ values were used to calculate the Q_10_ values (Table 2). Furthermore, the estimated values of E_a_ and A for the production of ethanol were used to illustrate the applicability of the Arrhenius equation for ethanol production as well as to illustrate the models obtained for the concentrations of ethanol as a function of time at 17, 21, 25, and 29°C (Figure 5). However, the effect of the inoculum could not be neglected. In fact, the volumetric production rates of all metabolites at 21°C calculated from the A and E_a_ values (131 mg L^−1^ h^−1^ for ethanol, 19.3 mg L^−1^ h^−1^ for lactic acid, 9.9 mg L^−1^ h^−1^ for acetic acid, 8.6 mg L^−1^ h^−1^ for glycerol, and 6.8 mg L^−1^ h^−1^ for mannitol) were lower than those reported for a similar fermentation process performed at the same temperature but inoculated with a different inoculum (Table 1).

**Table 2:** Estimated values for the pre-exponential factors (A), the activation energies (E_a_), and the Q_10_ values for the production kinetics of ethanol, lactic acid, acetic acid, glycerol, and mannitol during water kefir fermentation processes started with rinsed grains and incubated at 17, 21, 25, and 29°C.

**Metabolite**	**A (mg l^**−1**^ h^**−1**^)**	**E_**a**_ (kJ mol^**−1**^)**	**Q_**10**_**
Ethanol	(25.5 ± 49.7) 10^12^	63.6 ± 4.8	2.37 [2.08; 2.69]
Lactic acid	(113 ± 242) 10^12^	71.9 ± 5.3	2.64 [2.30; 3.04]
Acetic acid	(1.08 ± 1.54) 10^12^	62.2 ± 3.5	2.32 [2.11; 2.55]
Glycerol	(305 ± 776) 10^12^	76.3 ± 6.3	2.81 [2.38; 3.32]
Mannitol	(9.19 ± 12.92) 10^8^	45.8 ± 3.4	1.86 [1.70; 2.04]

*The results for A and E_a_ are presented as the mean ± standard error and the results for the Q_10_ values are presented as the mean and the 95% confidence interval*.

## Discussion

Water kefir fermentation is usually performed at room temperature with a backslopping time of 2–4 days, whereby the water kefir grains are rinsed during each backslopping step (Pidoux, 1989; Waldherr et al., 2010; Gulitz et al., 2011, 2013; Laureys and De Vuyst, 2014; Lynch et al., 2021). This study determined both short- and long-term influences of the backslopping time, rinsing off the water kefir grains during each backslopping step, and incubation temperature on the water kefir fermentation process.

Rinsing of the water kefir grains removed part of the substrates and microbial metabolites from the grains, resulting in lower substrate and metabolite concentrations in the liquors and higher pH values than when the grains were not rinsed. However, rinsing of the grains did not remove substantial numbers of LAB or yeasts and did not decrease the volumetric metabolite production rates significantly. The volumetric metabolite production rates were strongly influenced by the viable counts of the LAB and yeasts in the grain inoculum, as they were higher during the water kefir fermentation processes inoculated with non-rinsed grains from a grain inoculum with high viable counts of LAB and yeasts than during a similar fermentation process inoculated with non-rinsed grains from a grain inoculum with low viable counts of LAB and yeasts. This underlines the importance of the grain inoculum on the water kefir fermentation rate, confirming previous results (Laureys and De Vuyst, 2017; Laureys et al., 2021).

Short backslopping times resulted in low viable counts of AAB on the water kefir grains, which were even lower when the grains were rinsed during each backslopping step. Furthermore, rinsing of the grains during each backslopping step increased the relative abundance of *Lenl*. *hilgardii* and *S*. *cerevisiae* (both associated with the water kefir grains) and decreased the relative abundances of *D*. *bruxellensis* and *Liql*. *nagelii* (both associated with the water kefir liquors) (Laureys and De Vuyst, 2014, 2017).

Short backslopping times and rinsing of the grains during each backslopping step reduced the acidic stress of the microorganisms, which impacted the microbial species diversity during the water kefir fermentation processes studied. In fact, *Leuc*. *pseudomesenteroides* is sensitive to acidic stress (Ludwig et al., 2009) and was less abundant when the backslopping times were long or when the water kefir grains were not rinsed during each backslopping step. In contrast, *Oenococcus* species are generally not sensitive to acidic stress (Alegría et al., 2004) and *O. sicerae* was indeed present in higher relative abundances when the backslopping times were long and when the water kefir grains were not rinsed during each backslopping step. Furthermore, short backslopping times decreased the relative abundances of slow-growing microorganisms, such as *D. bruxellensis*, as the latter species grows slower than *S*. *cerevisiae* (Schifferdecker et al., 2014). The same mechanism may have caused the low relative abundances of *Leuc*. *pseudomesenteroides* at short backslopping times. The influence of the backslopping time is also well-known in backslopped sourdough productions (De Vuyst et al., 2017).

When the incubation temperature increased, the relative abundances of *Liql*. *mali* decreased and those of *Liql*. *nagelii* increased. In fact, it is known that the incubation temperature may influence the microbial species diversity during food fermentation, as encountered also, for example, in backslopped sourdough productions (Meroth et al., 2003; Vrancken et al., 2011; De Vuyst et al., 2017). The relative abundances of *Leuc*. *pseudomesenteroides* were highest at intermediate incubation temperatures (21–25°C), which is in agreement with the optimal growth temperature of ~20–30°C for *Leuconostoc* species (Ludwig et al., 2009) and the high relative abundance of particular *Leuconostoc* species at 23°C during backslopped wheat sourdough productions (Vrancken et al., 2011). The incubation temperature did not influence the yeast communities, which is in agreement with the optimal growth temperature of the yeasts found in this study (Kurtzman et al., 2011).

Overall, a shift in the microbial communities did not substantially influence the concentrations of the different metabolites produced, except for mannitol. High concentrations of mannitol coincided with high relative abundances of *Lenl*. *hilgardii*, an obligately heterofermentative LAB species (Ludwig et al., 2009) that can reduce fructose into mannitol (Zaunmüller et al., 2006).

Values of the pH higher than 3.4 ensured that the water kefir grain growth remained stable and high, as low pH values could decrease this growth (Laureys et al., 2019). The water kefir grain growth was slightly higher when the grains were rinsed during each backslopping step. This may be caused by the high pH values during these fermentation series, as the activity of glucansucrases is lower at low pH values (Waldherr et al., 2010) or by the high relative abundances of *Lenl*. *hilgardii* in these fermentation series, as this LAB species is thought to be responsible for the water kefir grain growth during fermentation (Pidoux, 1989; Waldherr et al., 2010). In fact, the main EPS-producing LAB species in the water kefir fermentation processes studied was *Lenl. hilgardii* and, hence, it is likely that this EPS production is related to the mass of the grains. However, the presence and relative abundance of *Lenl. hilgardii* or EPS-producing strains of other species do not always correspond with the water kefir grain growth, as has been reported before (Laureys and De Vuyst, 2017; Laureys et al., 2018, 2019). Additionally, *Liql*. *mali* and *Leuc*. *pseudomesenteroides* produced EPS from sucrose. These LAB species were more strongly associated with the water kefir liquors and their presence did not influence the water kefir grain growth, which has been shown before (Laureys and De Vuyst, 2017; Laureys et al., 2018, 2019). Furthermore, only a few *Liql*. *nagelii* isolates from the fermentation series with the lowest pH values produced EPS from sucrose. This was in line with a previous report that showed the presence of EPS-producing *Liql*. *nagelii* isolates only in water kefir fermentations with low pH values (Laureys and De Vuyst, 2017). This LAB species was not strongly associated with the grains and did not always produce EPS, indicating that it was probably not responsible for the water kefir grain growth.

The influence of the temperature on the volumetric production rates of ethanol, lactic acid, acetic acid, and glycerol was quantified by determining the parameters of the Arrhenius equation for each metabolite. The activation energy (E_a_) for the production of ethanol during water kefir fermentation was similar to the E_a_ of 65 kJ mol^−1^ for the production of ethanol by *S*. *cerevisiae* (Ortiz-Muñiz et al., 2010), the E_a_ of 69.5 kJ mol^−1^ for the production of ethanol by *D*. *bruxellensis* (Brandam et al., 2007), and the E_a_ of 64.3 kJ mol^−1^ for the production of ethanol during milk kefir fermentation (Zajšek and Goršek, 2010). The E_a_ for the production of lactic acid during water kefir fermentation was similar to the E_a_ of 71.5 kJ mol^−1^ for the production of lactic acid by *Lactobacillus delbrueckii* (a homofermentative LAB species) at pH 5.5 (Kempe et al., 1956), the E_a_ of 77–79 kJ mol^−1^ for the production of lactic acid by *Lacc*. *paracasei* at pH 6.0 (Adamberg et al., 2003), and the E_a_ of 84.7 kJ mol^−1^ for the production of lactic acid by *Lactobacillus amylovorus* at pH 5.4 (Messens et al., 2002).

## Conclusion

Rinsing of the water kefir grains during each backslopping step decreased the concentrations of metabolites and the relative abundances of liquor-associated microorganisms and increased the water kefir grain growth and the relative abundances of grain-associated microorganisms. However, the total number of yeasts and LAB remained similar, as well as the volumetric production rates for the different metabolites. Short backslopping times decreased the relative abundances of slow-growing microorganisms, and long backslopping times decreased the relative abundances of acid-sensitive microorganisms. The number of AAB decreased when the backslopping time was one day. The microbial communities were impacted by the incubation temperature, but this shift in microbial communities had only minor effects on the production of the different metabolites. The water kefir fermentation rate was mainly determined by the viable counts of the LAB and yeasts on the water kefir grain inoculum and by the incubation temperature, but not by rinsing of the water kefir grains.

## Data Availability Statement

The datasets presented in this study can be found in online repositories. The names of the repository/repositories and accession number(s) can be found in the article/Supplementary Material.

## Author Contributions

DL, LD, and FL designed the study, performed the experiments, acquired the experimental data, interpreted the data, and performed the statistical analyses. DL and PV performed the culture-dependent microbial species diversity analyses. DL, LD, and FL wrote the manuscript in consultation with PV. All authors provided critical revisions and approved the final version of the manuscript.

## Funding

The authors acknowledge the financial support of the Research Council of the Vrije Universiteit Brussel (SRP7, IRP2, and IOF2242 projects) and the Hercules Foundation (Grant no. UABR09004). DL was the recipient of a Ph.D. fellowship from the Vrije Universiteit Brussel.

## Conflict of Interest

The authors declare that the research was conducted in the absence of any commercial or financial relationships that could be construed as a potential conflict of interest.

## Publisher's Note

All claims expressed in this article are solely those of the authors and do not necessarily represent those of their affiliated organizations, or those of the publisher, the editors and the reviewers. Any product that may be evaluated in this article, or claim that may be made by its manufacturer, is not guaranteed or endorsed by the publisher.
