# Supplementary material for: Backslopping Time, Rinsing of the Grains During Backslopping, and Incubation Temperature Influence the Water Kefir Fermentation Process
Source: Front Microbiol. 2022 May 6;13:871550. doi: 10.3389/fmicb.2022.871550 (PMC9120925; doi:10.3389/fmicb.2022.871550)
Supplement: Supplementary file 1 [file Table_1.DOCX]

Supplementary Material

Figures

**Figure S1.** pH and water kefir grain growth of the eight series of water kefir fermentations differing in backslopping time and rinsing of the grains during each backslopping step, at the end of backslopping step 8 [backslopping time of 1 d with (1D-R, ○) or without rinsing (1D-NR, ●), backslopping time of 2 d with (2D-R, ◊) or without rinsing (2D-NR, ♦), backslopping time of 3 d with (3D-R, Δ) or without rinsing (3D-NR, ▲), and backslopping time of 4 d with (4D-R, □) or without rinsing (4D-NR, ■)] (top), and for the eight series of water kefir fermentations differing in incubation temperature and backslopping time [incubation temperature of 17 °C with a backslopping time of 3 d (17C-3D, ○) or 4 d (17C-4D, ●), incubation temperature of 21 °C with a backslopping time of 2 d (21C-2D, ◊) or 3 d (21C-3D, ♦), incubation temperature of 25 °C with a backslopping time of 2 d (25C-2D, Δ) or 3 d (25D-3D, ▲), and incubation temperature of 29 °C with a backslopping time of 1 d (29C-1D, □) or 2 d (29C-2D, ■)] (bottom). C, temperature; D, days of backslopping; R, rinsed; NR, non-rinsed.

Tables

**Table S1.** Characteristics of the eight series of water kefir fermentations differing in backslopping time and rinsing of the grains during each backslopping step [backslopping time of 1 d with (1D-R) or without rinsing (1D-NR), 2 d with (2D-R) or without rinsing (2D-NR), 3 d with (3D-R) or without rinsing (3D-NR), or 4 d with (4D-R) or without rinsing (4D-NR)] at the end of backslopping step 1. Significant differences (p < 0.05) between the series are indicated with different superscripts (a, b, c, d, e, and f). D, days of backslopping; R, rinsed; NR, non-rinsed.

| Characteristic | 1D-R | 1D-NR | 2D-R | 2D-NR | 3D-R | 3D-NR | 4D-R | 4D-NR |
| --- | --- | --- | --- | --- | --- | --- | --- | --- |
| Water kefir grain growth (%) | 51.8 ± 3.9^c^ | 51.7 ± 5.3 ^c^ | 57.9 ± 2.1 ^ab^ | 56.7 ± 1.3 ^bc^ | 57.9 ± 2.6 ^ab^ | 59.7 ± 2.2 ^ab^ | 60.3 ± 1.7 ^ab^ | 62.4 ± 2.7 ^a^ |
| pH | 3.91 ± 0.04 ^a^ | 3.77 ± 0.07 ^b^ | 3.56 ± 0.03 ^c^ | 3.52 ± 0.04 ^cd^ | 3.48 ± 0.03 ^de^ | 3.41 ± 0.03 ^f^ | 3.44 ± 0.02 ^ef^ | 3.40 ± 0.01 ^f^ |
| Sucrose (g l^-1^) | 3.4 ± 0.9 ^b^ | 4.3 ± 0.7 ^a^ | 1.0 ± 0.2 ^c^ | 1.0 ± 0.1 ^c^ | 0.9 ± 0.3 ^c^ | 1.1 ± 0.4 ^c^ | 1.2 ± 0.2 ^c^ | 0.9 ± 0.3 ^c^ |
| Glucose (g l^-1^) | 4.4 ± 0.6 ^a^ | 4.2 ± 0.7 ^a^ | 1.8 ± 0.8 ^b^ | 1.0 ± 0.4 ^c^ | 0.4 ± 0.4 ^cd^ | 0.0 ± 0.0 ^d^ | 0.0 ± 0.0 ^d^ | 0.0 ± 0.0 ^d^ |
| Fructose (g l^-1^) | 16.9 ± 2.2 ^a^ | 16.4 ± 1.6 ^a^ | 12.8 ± 4.2 ^ab^ | 9.9 ± 1.1 ^bc^ | 6.1 ± 4.1 ^c^ | 1.8 ± 1.9 ^d^ | 0.2 ± 0.1 ^d^ | 0.1 ± 0.0 ^d^ |
| Total carbohydrates (g l^-1^) | 24.7 ± 3.7 ^a^ | 24.9 ± 2.6 ^a^ | 15.6 ± 5.1 ^b^ | 11.8 ± 1.4 ^bc^ | 7.3 ± 4.7 ^cd^ | 2.9 ± 2.3 ^de^ | 1.4 ± 0.2 ^e^ | 1.0 ± 0.3 ^e^ |
| Ethanol (g l^-1^) | 6.0 ± 0.2 ^f^ | 9.1 ± 0.9 ^e^ | 11.7 ± 0.2 ^d^ | 15.3 ± 0.8 ^c^ | 15.5 ± 2.1 ^c^ | 19.9 ± 0.9 ^ab^ | 18.7 ± 0.2 ^b^ | 21.5 ± 0.7 ^a^ |
| Lactic acid (g l^-1^) | 1.04 ± 0.13 ^e^ | 1.34 ± 0.11 ^d^ | 1.89 ± 0.07 ^c^ | 2.26 ± 0.12 ^b^ | 2.35 ± 0.36 ^b^ | 2.87 ± 0.13 ^a^ | 2.91 ± 0.12 ^a^ | 3.11 ± 0.05 ^a^ |
| Acetic acid (g l^-1^) | 0.41 ± 0.01 ^f^ | 0.56 ± 0.02 ^e^ | 0.74 ± 0.03 ^d^ | 0.92 ± 0.02 ^c^ | 0.96 ± 0.10 ^c^ | 1.15 ± 0.06 ^b^ | 1.15 ± 0.04 ^b^ | 1.30 ± 0.03 ^a^ |
| Glycerol (g l^-1^) | 0.95 ± 0.09 ^d^ | 1.16 ± 0.10 ^cd^ | 1.60 ± 0.54 ^bcd^ | 1.73 ± 0.22 ^bc^ | 1.94 ± 0.49 ^ab^ | 2.49 ± 0.82 ^a^ | 1.88 ± 0.15 ^abc^ | 2.60 ± 0.50 ^a^ |
| Mannitol (g l^-1^) | 0.23 ± 0.11 ^c^ | 0.19 ± 0.01 ^c^ | 0.36 ± 0.11 ^bc^ | 0.34 ± 0.07 ^bc^ | 0.53 ± 0.18 ^ab^ | 0.51 ± 0.14 ^ab^ | 0.48 ± 0.09 ^ab^ | 0.58 ± 0.17 ^a^ |
| 2-Methyl-1-propanol (mg l^-1^) | 4.4 ± 0.5 ^f^ | 6.1 ± 0.3 ^e^ | 7.5 ± 0.2 ^d^ | 9.0 ± 1.0 ^bc^ | 8.4 ± 1.4 ^cd^ | 10.2 ± 0.3 ^a^ | 9.7 ± 0.1 ^ab^ | 10.7 ± 0.4 ^a^ |
| Isoamyl alcohol (mg l^-1^) | 18.8 ± 1.5 ^e^ | 25.9 ± 0.5 ^d^ | 33.6 ± 0.7 ^c^ | 38.8 ± 1.5 ^b^ | 40.7 ± 3.2 ^b^ | 43.8 ± 0.4 ^a^ | 43.5 ± 0.8 ^a^ | 45.9 ± 0.6 ^a^ |
| Ethyl acetate (mg l^-1^) | 2.6 ± 0.3 ^f^ | 5.8 ± 0.4 ^e^ | 8.1 ± 0.9 ^d^ | 11.0 ± 1.4 ^c^ | 12.2 ± 0.9 ^c^ | 15.9 ± 1.1 ^b^ | 17.5 ± 2.2 ^b^ | 20.8 ± 0.7 ^a^ |
| Isoamyl acetate (mg l^-1^) | 0.073 ± 0.003 ^d^ | 0.089 ± 0.006 ^cd^ | 0.094 ± 0.004 ^bc^ | 0.108 ± 0.015 ^ab^ | 0.101 ± 0.018 ^bc^ | 0.126 ± 0.012 ^a^ | 0.111 ± 0.009 ^ab^ | 0.123 ± 0.007 ^a^ |
| Ethyl hexanoate (mg l^-1^) | 0.16 ± 0.01 ^c^ | 0.17 ± 0.01 ^bc^ | 0.18 ± 0.01 ^ab^ | 0.20 ± 0.01 ^a^ | 0.18 ± 0.01 ^ab^ | 0.20 ± 0.01 ^a^ | 0.19 ± 0.01 ^a^ | 0.19 ± 0.01 ^a^ |
| Ethyl octanoate (mg l^-1^) | 0.22 ± 0.04 ^c^ | 0.26 ± 0.05 ^c^ | 0.30 ± 0.09 ^bc^ | 0.33 ± 0.09 ^abc^ | 0.35 ± 0.06 ^abc^ | 0.42 ± 0.10 ^ab^ | 0.41 ± 0.09 ^ab^ | 0.44 ± 0.08 ^a^ |
| Glycerol/ethanol (mmol/mol) | 0.080 ± 0.009 | 0.064 ± 0.006 | 0.068 ± 0.022 | 0.056 ± 0.005 | 0.064 ± 0.020 | 0.062 ± 0.020 | 0.050 ± 0.004 | 0.060 ± 0.011 |
| Lactic acid/ethanol (mmol/mol) | 0.089 ± 0.009 ^a^ | 0.076 ± 0.002 ^c^ | 0.083 ± 0.001 ^ab^ | 0.075 ± 0.001 ^c^ | 0.078 ± 0.002 ^bc^ | 0.074 ± 0.001 ^c^ | 0.080 ± 0.003 ^bc^ | 0.074 ± 0.003 ^c^ |
| Acetic acid/ethanol (mmol/mol) | 0.052 ± 0.001 ^a^ | 0.048 ± 0.003 ^bc^ | 0.049 ± 0.002 ^ab^ | 0.046 ± 0.002 ^bc^ | 0.048 ± 0.003 ^bc^ | 0.044 ± 0.001 ^c^ | 0.047 ± 0.001 ^bc^ | 0.047 ± 0.002 ^bc^ |
| Acetic acid/lactic acid (mmol/mol) | 0.59 ± 0.06 | 0.63 ± 0.03 | 0.59 ± 0.03 | 0.61 ± 0.02 | 0.62 ± 0.04 | 0.60 ± 0.02 | 0.59 ± 0.04 | 0.63 ± 0.01 |
| D-Lactic acid (% of total) | 45.0 ± 3.6 | 43.9 ± 1.0 | 44.7 ± 0.6 | 45.0 ± 0.4 | 45.6 ± 0.3 | 45.8 ± 0.4 | 46.1 ± 0.2 | 45.9 ± 0.2 |

**Table S2.** Characteristics of the eight series of water kefir fermentations differing in backslopping time and rinsing of the grains during each backslopping step [backslopping time of 1 d with (1D-R) or without rinsing (1D-NR), 2 d with (2D-R) or without rinsing (2D-NR), 3 d with (3D-R) or without rinsing (3D-NR), and 4 d with (4D-R) or without rinsing (4D-NR)] at the end of backslopping step 8. Significant differences (p < 0.05) between the series are indicated with different superscripts (a, b, c, d, and e). D, days of backslopping; R, rinsed; NR, non-rinsed.

| Characteristic | 1D-R | 1D-NR | 2D-R | 2D-NR | 3D-R | 3D-NR | 4D-R | 4D-NR |
| --- | --- | --- | --- | --- | --- | --- | --- | --- |
| Yeasts [log (cfu g^-1^)] | 7.6 ± 0.2 ^ab^ | 7.5 ± 0.1 ^bcd^ | 7.4 ± 0.1 ^d^ | 7.5 ± 0.1 ^bcd^ | 7.5 ± 0.1 ^cd^ | 7.6 ± 0.1 ^bc^ | 7.6 ± 0.1 ^ab^ | 7.7 ± 0.1 ^a^ |
| Lactic acid bacteria [log (cfu g^-1^)] | 8.6 ± 0.1 | 8.6 ± 0.1 | 8.4 ± 0.1 | 8.3 ± 0.1 | 8.5 ± 0.1 | 8.6 ± 0.1 | 8.5 ± 0.2 | 8.5 ± 0.2 |
| Acetic acid bacteria [log (cfu g^-1^)] | BQL | 2.8 ± 1.5 ^b^ | 4.6 ± 0.2 ^a^ | 4.6 ± 0.2 ^a^ | 4.3 ± 0.2 ^a^ | 4.6 ± 0.1 ^a^ | 4.3 ± 0.9 ^a^ | 4.0 ± 1.0 ^ab^ |
| Lactic acid bacteria/yeasts (cfu/cfu) | 10.0 ± 4.9 | 13.3 ± 6.3 | 9.8 ± 1.9 | 7.3 ± 2.4 | 11.8 ± 1.1 | 11.8 ± 0.8 | 8.4 ± 3.8 | 6.4 ± 2.2 |
| Water kefir grain growth (%) | 55.7 ± 2.7 ^a^ | 41.8 ± 1.0 ^e^ | 52.5 ± 1.5 ^ab^ | 45.6 ± 0.8 ^de^ | 54.4 ± 3.3 ^a^ | 48.2 ± 1.2 ^cd^ | 50.2 ± 2.4 ^bc^ | 44.5 ± 3.3 ^de^ |
| Water kefir grain dry mass (%) | 15.6 ± 0.3 ^c^ | 17.1 ± 0.1 ^a^ | 15.5 ± 0.3 ^c^ | 16.4 ± 0.1 ^b^ | 14.9 ± 0.3 ^d^ | 15.4 ± 0.2 ^c^ | 14.4 ± 0.4 ^e^ | 14.5 ± 0.3 ^de^ |
| pH | 3.94 ± 0.03 ^a^ | 3.95 ± 0.09 ^a^ | 3.69 ± 0.04 ^b^ | 3.69 ± 0.07 ^b^ | 3.55 ± 0.05 ^c^ | 3.52 ± 0.03 ^c^ | 3.46 ± 0.06 ^cd^ | 3.40 ± 0.02 ^d^ |
| Sucrose (g l^-1^) | 2.4 ± 0.3 ^c^ | 10.1 ± 0.6 ^a^ | 1.6 ± 0.1 ^d^ | 4.0 ± 0.4 ^b^ | 1.5 ± 0.1 ^d^ | 1.6 ± 0.1 ^d^ | 1.4 ± 0.1 ^d^ | 1.4 ± 0.1 ^d^ |
| Glucose (g l^-1^) | 5.3 ± 0.2 ^b^ | 6.4 ± 0.3 ^a^ | 4.8 ± 0.8 ^b^ | 5.5 ± 0.4 ^b^ | 2.5 ± 0.2 ^d^ | 3.4 ± 0.4 ^c^ | 0.8 ± 0.9 ^e^ | 0.6 ± 0.4 ^e^ |
| Fructose (g l^-1^) | 23.8 ± 0.7 ^a^ | 24.0 ± 1.6 ^a^ | 23.3 ± 1.3 ^ab^ | 23.7 ± 1.1 ^a^ | 18.1 ± 1.2 ^c^ | 19.5 ± 1.0 ^bc^ | 10.2 ± 5.4 ^d^ | 10.0 ± 2.5 ^d^ |
| Total carbohydrates (g l^-1^) | 31.5 ± 0.6 ^b^ | 40.5 ± 2.2 ^a^ | 29.7 ± 2.0 ^b^ | 33.2 ± 1.6 ^b^ | 22.2 ± 1.5 ^c^ | 24.4 ± 1.3 ^c^ | 12.5 ± 6.4 ^d^ | 12.0 ± 3.0 ^d^ |
| Ethanol (g l^-1^) | 5.5 ± 0.1 ^e^ | 5.2 ± 1.4 ^e^ | 7.2 ± 1.0 ^de^ | 7.9 ± 1.1 ^de^ | 10.2 ± 0.9 ^cd^ | 12.2 ± 0.5 ^bc^ | 14.8 ± 3.1 ^b^ | 20.1 ± 3.1 ^a^ |
| Lactic acid (g l^-1^) | 0.87 ± 0.03 ^e^ | 0.91 ± 0.21 ^e^ | 1.25 ± 0.10 ^d^ | 1.32 ± 0.08 ^d^ | 1.68 ± 0.12 ^c^ | 1.87 ± 0.04 ^c^ | 2.20 ± 0.27 ^b^ | 2.54 ± 0.31 ^a^ |
| Acetic acid (g l^-1^) | 0.34 ± 0.01 ^d^ | 0.37 ± 0.04 ^d^ | 0.65 ± 0.08 ^c^ | 0.65 ± 0.05 ^c^ | 0.94 ± 0.06 ^b^ | 0.93 ± 0.09 ^b^ | 1.17 ± 0.11 ^a^ | 1.17 ± 0.02 ^a^ |
| Glycerol (g l^-1^) | 0.72 ± 0.04 ^de^ | 0.61 ± 0.06 ^e^ | 0.89 ± 0.11 ^d^ | 0.84 ± 0.05 ^de^ | 1.22 ± 0.07 ^c^ | 1.27 ± 0.07 ^c^ | 1.64 ± 0.27 ^b^ | 2.02 ± 0.30 ^a^ |
| Mannitol (g l^-1^) | 0.20 ± 0.08 ^cd^ | 0.15 ± 0.10 ^d^ | 0.31 ± 0.09 ^cd^ | 0.16 ± 0.05 ^d^ | 0.66 ± 0.21 ^b^ | 0.35 ± 0.10 ^c^ | 0.84 ± 0.02 ^a^ | 0.57 ± 0.02 ^b^ |
| 2-Methyl-1-propanol (mg l^-1^) | 4.6 ± 0.6 ^b^ | 3.9 ± 0.6 ^b^ | 4.5 ± 0.7 ^b^ | 4.3 ± 0.7 ^b^ | 4.4 ± 0.7 ^b^ | 4.8 ± 0.4 ^b^ | 5.6 ± 2.1 ^ab^ | 7.2 ± 1.2 ^a^ |
| Isoamyl alcohol (mg l^-1^) | 14.2 ± 1.7 ^e^ | 14.2 ± 0.1 ^e^ | 20.8 ± 3.5 ^cd^ | 20.0 ± 2.4 ^de^ | 24.0 ± 3.6 ^cd^ | 25.9 ± 1.1 ^bc^ | 31.5 ± 6.6 ^ab^ | 36.7 ± 3.6 ^a^ |
| Ethyl acetate (mg l^-1^) | 2.6 ± 0.2 ^d^ | 2.4 ± 0.8 ^d^ | 6.4 ± 0.7 ^c^ | 6.6 ± 0.6 ^c^ | 13.4 ± 2.1 ^a^ | 9.2 ± 2.0 ^b^ | 13.6 ± 2.6 ^a^ | 10.8 ± 0.6 ^b^ |
| Isoamyl acetate (mg l^-1^) | 0.068 ± 0.001 ^c^ | 0.066 ± 0.009 ^c^ | 0.068 ± 0.003 ^c^ | 0.069 ± 0.003 ^c^ | 0.075 ± 0.006 ^bc^ | 0.080 ± 0.007 ^bc^ | 0.086 ± 0.015 ^b^ | 0.110 ± 0.017 ^a^ |
| Ethyl hexanoate (mg l^-1^) | 0.16 ± 0.01 ^bc^ | 0.16 ± 0.02 ^bc^ | 0.15 ± 0.01 ^c^ | 0.16 ± 0.01 ^bc^ | 0.17 ± 0.01 ^bc^ | 0.18 ± 0.02 ^bc^ | 0.18 ± 0.01 ^b^ | 0.23 ± 0.03 ^a^ |
| Ethyl octanoate (mg l^-1^) | 0.32 ± 0.13 ^bc^ | 0.29 ± 0.06 ^c^ | 0.34 ± 0.06 ^bc^ | 0.38 ± 0.09 ^bc^ | 0.47 ± 0.07 ^abc^ | 0.51 ± 0.22 ^abc^ | 0.54 ± 0.18 ^ab^ | 0.65 ± 0.13 ^a^ |
| Glycerol/ethanol (mmol/mol) | 0.066 ± 0.005 ^a^ | 0.060 ± 0.009 ^abc^ | 0.061 ± 0.001 ^ab^ | 0.054 ± 0.004 ^cd^ | 0.060 ± 0.002 ^abc^ | 0.052 ± 0.001 ^d^ | 0.056 ± 0.003 ^bcd^ | 0.050 ± 0.004 ^d^ |
| Lactic acid/ethanol (mmol/mol) | 0.082 ± 0.002 ^abc^ | 0.090 ± 0.003 ^a^ | 0.089 ± 0.008 ^a^ | 0.086 ± 0.007 ^ab^ | 0.084 ± 0.002 ^abc^ | 0.078 ± 0.002 ^bc^ | 0.077 ± 0.007 ^c^ | 0.065 ± 0.004 ^d^ |
| Acetic acid/ethanol (mmol/mol) | 0.047 ± 0.002 ^bc^ | 0.056 ± 0.010 ^abc^ | 0.071 ± 0.016 ^a^ | 0.064 ± 0.013 ^ab^ | 0.071 ± 0.012 ^a^ | 0.059 ± 0.005 ^abc^ | 0.062 ± 0.008 ^abc^ | 0.045 ± 0.006 ^c^ |
| Acetic acid/lactic acid (mmol/mol) | 0.58 ± 0.04 ^c^ | 0.62 ± 0.09 ^bc^ | 0.79 ± 0.12 ^a^ | 0.74 ± 0.10 ^ab^ | 0.84 ± 0.12 ^a^ | 0.75 ± 0.06 ^ab^ | 0.80 ± 0.04 ^a^ | 0.70 ± 0.07 ^abc^ |
| D-lactic acid (% of total) | 42.1 ± 0.3 ^cd^ | 40.7 ± 2.0 ^d^ | 42.7 ± 0.8 ^bc^ | 44.2 ± 0.7 ^ab^ | 44.7 ± 0.9 ^a^ | 44.9 ± 0.6 ^a^ | 45.7 ± 0.5 ^a^ | 45.1 ± 0.3 ^a^ |

**Table S3.** Characteristics of the eight series of water kefir fermentations differing in incubation temperature and backslopping time [incubation temperature of 17 °C with a backslopping time of 3 d (17C-3D) or 4 d (17C-4D), 21 °C with a backslopping time of 2 d (21C-2D) or 3 d (21C-3D), 25 °C with a backslopping time of 2 d (25C-2D) or 3 d (25D-3D), and 29 °C with a backslopping time of 1 d (29C-1D) or 2 d (29C-2D)] at the end of backslopping step 1. Significant differences (p < 0.05) between the series are indicated with different superscripts (a, b, c, d, and e). C, temperature; D, days of backslopping.

| Characteristic | 17C-3D | 17C-4D | 21C-2D | 21C-3D | 25C-2D | 25C-3D | 29C-1D | 29C-2D |
| --- | --- | --- | --- | --- | --- | --- | --- | --- |
| Water kefir grain growth (%) | 53.2 ± 0.8 ^c^ | 45.3 ± 2.1 ^e^ | 49.2 ± 2.2 ^d^ | 57.9 ± 0.9 ^ab^ | 59.7 ± 3.0 ^a^ | 59.5 ± 3.3 ^a^ | 55.2 ± 2.3 ^bc^ | 59.5 ± 2.2 ^a^ |
| pH | 3.80 ± 0.10 ^a^ | 3.59 ± 0.04 ^cd^ | 3.73 ± 0.14 ^ab^ | 3.65 ± 0.05 ^bc^ | 3.63 ± 0.05 ^bc^ | 3.48 ± 0.05 ^de^ | 3.84 ± 0.08 ^a^ | 3.44 ± 0.06 ^e^ |
| Sucrose (g l^-1^) | 1.7 ± 0.1 | 1.9 ± 0.1 | 1.6 ± 0.1 | 1.6 ± 0.1 | 1.5 ± 0.1 | 1.7 ± 0.1 | 1.9 ± 0.3 | 1.0 ± 0.8 |
| Glucose (g l^-1^) | 5.4 ± 1.0 ^a^ | 2.7 ± 0.6 ^c^ | 4.7 ± 1.0 ^ab^ | 3.0 ± 0.5 ^c^ | 3.5 ± 0.3 ^bc^ | 0.8 ± 0.6 ^d^ | 5.7 ± 0.5 ^a^ | 0.5 ± 0.7 ^d^ |
| Fructose (g l^-1^) | 24.5 ± 2.8 ^ab^ | 18.2 ± 1.6 ^c^ | 22.7 ± 2.2 ^abc^ | 19.4 ± 0.7 ^bc^ | 20.7 ± 0.8 ^abc^ | 12.1 ± 3.9 ^d^ | 24.9 ± 1.3 ^a^ | 7.5 ± 6.9 ^d^ |
| Total carbohydrates (g l^-1^) | 31.6 ± 3.9 ^a^ | 22.8 ± 2.2 ^b^ | 29.0 ± 3.3 ^ab^ | 24.0 ± 1.1 ^b^ | 25.7 ± 1.1 ^ab^ | 14.6 ± 4.4 ^c^ | 32.5 ± 2.0 ^a^ | 9.0 ± 8.3 ^c^ |
| Ethanol (g l^-1^) | 6.8 ± 0.8 ^d^ | 10.8 ± 0.8 ^b^ | 7.8 ± 1.4 ^cd^ | 10.1 ± 0.5 ^b^ | 9.3 ± 0.8 ^bc^ | 14.0 ± 1.7 ^a^ | 6.2 ± 0.8 ^d^ | 14.6 ± 1.8 ^a^ |
| Lactic acid (g l^-1^) | 1.11 ± 0.09 ^d^ | 1.74 ± 0.13 ^b^ | 1.33 ± 0.25 ^cd^ | 1.67 ± 0.08 ^b^ | 1.64 ± 0.06 ^bc^ | 2.35 ± 0.30 ^a^ | 1.13 ± 0.17 ^d^ | 2.52 ± 0.19 ^a^ |
| Acetic acid (g l^-1^) | 0.56 ± 0.03 ^d^ | 0.81 ± 0.04 ^c^ | 0.61 ± 0.05 ^d^ | 0.81 ± 0.06 ^c^ | 0.82 ± 0.03 ^c^ | 1.16 ± 0.16 ^a^ | 0.54 ± 0.04 ^d^ | 1.02 ± 0.03 ^b^ |
| Glycerol (g l^-1^) | 0.78 ± 0.05 ^c^ | 1.07 ± 0.10 ^b^ | 0.87 ± 0.12 ^c^ | 1.08 ± 0.03 ^b^ | 1.12 ± 0.06 ^b^ | 1.49 ± 0.14 ^a^ | 0.78 ± 0.03 ^c^ | 1.46 ± 0.09 ^a^ |
| Mannitol (g l^-1^) | 0.39 ± 0.01 ^d^ | 0.60 ± 0.07 ^b^ | 0.37 ± 0.03 ^de^ | 0.52 ± 0.03 ^bc^ | 0.51 ± 0.02 ^c^ | 0.71 ± 0.07 ^a^ | 0.31 ± 0.02 ^e^ | 0.58 ± 0.04 ^bc^ |
| 2-Methyl-1-propanol (mg l^-1^) | 3.8 ± 0.2 ^d^ | 4.8 ± 0.4 ^cd^ | 4.8 ± 0.7 ^cd^ | 5.3 ± 0.4 ^bc^ | 4.9 ± 0.6 ^cd^ | 6.4 ± 0.9 ^b^ | 3.8 ± 0.7 ^d^ | 8.0 ± 1.2 ^a^ |
| Isoamyl alcohol (mg l^-1^) | 18.1 ± 2.0 ^de^ | 25.7 ± 2.5 ^bc^ | 21.2 ± 3.1 ^cd^ | 27.0 ± 3.7 ^b^ | 23.7 ± 2.5 ^bc^ | 33.7 ± 3.8 ^a^ | 16.1 ± 1.5 ^e^ | 36.3 ± 3.1 ^a^ |
| Ethyl acetate (mg l^-1^) | 4.1 ± 0.8 ^c^ | 9.5 ± 2.3 ^b^ | 4.9 ± 1.1 ^c^ | 9.7 ± 0.4 ^b^ | 8.5 ± 2.5 ^b^ | 14.4 ± 1.4 ^a^ | 3.2 ± 0.8 ^c^ | 10.6 ± 0.6 ^b^ |
| Isoamyl acetate (mg l^-1^) | 0.066 ± 0.005 ^cd^ | 0.080 ± 0.006 ^a^ | 0.072 ± 0.007 ^abc^ | 0.073 ± 0.005 ^abc^ | 0.068 ± 0.003 ^bcd^ | 0.079 ± 0.005 ^a^ | 0.061 ± 0.002 ^d^ | 0.075 ± 0.002 ^ab^ |
| Ethyl hexanoate (mg l^-1^) | 0.15 ± 0.01 ^c^ | 0.17 ± 0.01 ^a^ | 0.16 ± 0.01 ^bc^ | 0.17 ± 0.01 ^ab^ | 0.15 ± 0.01 ^cd^ | 0.16 ± 0.01 ^bc^ | 0.14 ± 0.01 ^d^ | 0.15 ± 0.01 ^c^ |
| Ethyl octanoate (mg l^-1^) | 0.38 ± 0.14 | 0.52 ± 0.13 | 0.33 ± 0.07 | 0.41 ± 0.11 | 0.30 ± 0.09 | 0.44 ± 0.07 | 0.25 ± 0.04 | 0.42 ± 0.01 |
| Glycerol/ethanol (mmol/mol) | 0.058 ± 0.003 ^bc^ | 0.050 ± 0.003 ^d^ | 0.056 ± 0.003 ^bc^ | 0.054 ± 0.002 ^cd^ | 0.060 ± 0.002 ^ab^ | 0.053 ± 0.002 ^cd^ | 0.063 ± 0.005 ^a^ | 0.050 ± 0.003 ^b^ |
| Lactic acid/ethanol (mmol/mol) | 0.084 ± 0.003 | 0.083 ± 0.008 | 0.087 ± 0.002 | 0.085 ± 0.002 | 0.090 ± 0.004 | 0.086 ± 0.003 | 0.093 ± 0.003 | 0.088 ± 0.005 |
| Acetic acid/ethanol (mmol/mol) | 0.064 ± 0.004 ^ab^ | 0.057 ± 0.004 ^bc^ | 0.061 ± 0.006 ^abc^ | 0.062 ± 0.005 ^abc^ | 0.068 ± 0.007 ^a^ | 0.063 ± 0.001 ^ab^ | 0.067 ± 0.004 ^a^ | 0.054 ± 0.006 ^c^ |
| Acetic acid/lactic acid (mmol/mol) | 0.76 ± 0.03 ^a^ | 0.70 ± 0.02 ^a^ | 0.70 ± 0.08 ^a^ | 0.73 ± 0.04 ^a^ | 0.76 ± 0.05 ^a^ | 0.74 ± 0.03 ^a^ | 0.72 ± 0.06 ^a^ | 0.61 ± 0.03 ^b^ |
| D-lactic acid (% of total) | 42.3 ± 0.9 ^d^ | 42.0 ± 0.7 ^d^ | 43.9 ± 1.0 ^bc^ | 45.1 ± 0.6 ^ab^ | 45.4 ± 0.4 ^a^ | 45.8 ± 0.4 ^a^ | 42.7 ± 1.3 ^cd^ | 45.5 ± 0.6 ^a^ |

**Table S4.** Characteristics of the eight series of water kefir fermentations differing in incubation temperature and backslopping time [incubation temperature of 17 °C with a backslopping time of 3 d (17C-3D) or 4 d (17C-4D), 21 °C with a backslopping time of 2 d (21C-2D) or 3 d (21C-3D), 25 °C with a backslopping time of 2 d (25C-2D) or 3 d (25D-3D), and 29 °C with a backslopping time of 1 d (29C-1D) or 2 d (29C-2D)] at the end of backslopping step 8. Significant differences (p < 0.05) between the series are indicated with different superscripts (a, b, c, d, and e). C, temperature; D, days of backslopping.

| Characteristic | 17C-3D | 17C-4D | 21C-2D | 21C-3D | 25C-2D | 25C-3D | 29C-1D | 29C-2D |
| --- | --- | --- | --- | --- | --- | --- | --- | --- |
| Yeasts [log (cfu g^-1^)] | 7.3 ± 0.1 ^c^ | 7.6 ± 0.1 ^a^ | 7.5 ± 0.1 ^ab^ | 7.3 ± 0.1 ^c^ | 7.4 ± 0.2 ^bc^ | 7.3 ± 0.1 ^bc^ | 7.5 ± 0.1 ^ab^ | 7.4 ± 0.1 ^bc^ |
| Lactic acid bacteria [log (cfu g^-1^)] | 8.5 ± 0.1 | 8.5 ± 0.2 | 8.5 ± 0.2 | 8.5 ± 0.1 | 8.5 ± 0.2 | 8.3 ± 0.2 | 8.4 ± 0.1 | 8.5 ± 0.1 |
| Acetic acid bacteria [log (cfu g^-1^)] | 5.0 ± 0.4 | 4.5 ± 0.4 | 5.1 ± 0.2 | 4.6 ± 0.1 | 5.0 ± 0.6 | 4.8 ± 0.5 | 4.6 ± 0.1 | 4.7 ± 0.2 |
| Lactic acid bacteria/yeasts (cfu/cfu) | 18.7 ± 2.4 ^a^ | 8.6 ± 2.9 ^b^ | 10.7 ± 4.2 ^b^ | 18.8 ± 4.7 ^a^ | 13.8 ± 6.5 ^ab^ | 10.9 ± 3.1 ^b^ | 8.0 ± 2.2 ^b^ | 11.8 ± 2.1 ^b^ |
| Water kefir grain growth (%) | 51.8 ± 3.1 ^[a^ | 50.6 ± 0.7 ^ab^ | 53.6 ± 2.7 ^a^ | 53.4 ± 1.6 ^a^ | 52.9 ± 2.1 ^a^ | 53.6 ± 1.2 ^a^ | 53.0 ± 0.5 ^a^ | 47.8 ± 3.0 ^b^ |
| Water kefir grain dry mass (%) | 15.4 ± 0.5 ^ab^ | 14.9 ± 0.3 ^bc^ | 15.3 ± 0.3 ^abc^ | 14.6 ± 0.4 ^c^ | 14.8 ± 0.6 ^bc^ | 13.8 ± 0.4 ^d^ | 15.9 ± 0.4 ^a^ | 15.0 ± 0.4 ^bc^ |
| pH | 3.71 ± 0.08 ^b^ | 3.63 ± 0.09 ^bc^ | 3.67 ± 0.05 ^bc^ | 3.57 ± 0.02 ^cd^ | 3.61 ± 0.13 ^bc^ | 3.44 ± 0.04 ^e^ | 3.85 ± 0.04 ^a^ | 3.47 ± 0.06 ^de^ |
| Sucrose (g l^-1^) | 2.0 ± 0.2 ^ab^ | 1.9 ± 0.1 ^ab^ | 1.8 ± 0.1 ^abc^ | 1.7 ± 0.1 ^bc^ | 1.6 ± 0.1 ^bc^ | 1.4 ± 0.1 ^c^ | 2.2 ± 0.5 ^a^ | 1.4 ± 0.5 ^c^ |
| Glucose (g l^-1^) | 4.2 ± 1.6 ^bc^ | 3.1 ± 1.1 ^bcd^ | 4.5 ± 0.6 ^b^ | 2.4 ± 0.5 ^cde^ | 3.2 ± 2.0 ^bcd^ | 1.0 ± 0.8 ^e^ | 6.6 ± 0.7 ^a^ | 1.6 ± 1.0 ^de^ |
| Fructose (g l^-1^) | 22.5 ± 3.8 ^ab^ | 20.3 ± 2.9 ^ab^ | 23.9 ± 1.7 ^ab^ | 18.0 ± 1.6 ^bc^ | 19.9 ± 6.1 ^b^ | 11.7 ± 4.3 ^d^ | 26.3 ± 0.9 ^a^ | 13.1 ± 3.9 ^cd^ |
| Total carbohydrates (g l^-1^) | 28.6 ± 5.5 ^ab^ | 25.3 ± 3.9 ^b^ | 30.1 ± 2.3 ^ab^ | 22.1 ± 2.1 ^bc^ | 24.7 ± 8.2 ^b^ | 14.0 ± 5.2 ^c^ | 35.1 ± 2.0 ^a^ | 16.1 ± 5.2 ^c^ |
| Ethanol (g l^-1^) | 7.5 ± 2.4 ^bcd^ | 9.6 ± 1.9 ^bc^ | 6.5 ± 1.0 ^cd^ | 10.5 ± 0.8 ^b^ | 9.1 ± 3.0 ^bc^ | 14.1 ± 2.3 ^a^ | 4.9 ± 0.4 ^d^ | 14.1 ± 1.5 ^a^ |
| Lactic acid (g l^-1^) | 1.54 ± 0.35 ^bcd^ | 1.59 ± 0.23 ^bc^ | 1.42 ± 0.17 ^cd^ | 1.92 ± 0.15 ^b^ | 1.83 ± 0.36 ^bc^ | 2.43 ± 0.34 ^a^ | 1.08 ± 0.08 ^d^ | 2.47 ± 0.26 ^a^ |
| Acetic acid (g l^-1^) | 0.86 ± 0.15 ^c^ | 0.86 ± 0.06 ^c^ | 0.85 ± 0.09 ^c^ | 1.07 ± 0.10 ^b^ | 1.06 ± 0.10 ^b^ | 1.36 ± 0.15 ^a^ | 0.62 ± 0.03 ^d^ | 1.13 ± 0.16 ^b^ |
| Glycerol (g l^-1^) | 0.92 ± 0.21 ^cd^ | 1.22 ± 0.29 ^c^ | 0.91 ± 0.11 ^cd^ | 1.26 ± 0.18 ^bc^ | 1.26 ± 0.33 ^bc^ | 1.61 ± 0.15 ^ab^ | 0.80 ± 0.08 ^d^ | 1.77 ± 0.19 ^a^ |
| Mannitol (g l^-1^) | 0.86 ± 0.14 ^bc^ | 0.64 ± 0.14 ^c^ | 1.38 ± 0.28 ^a^ | 1.18 ± 0.38 ^ab^ | 1.40 ± 0.22 ^a^ | 1.39 ± 0.31 ^a^ | 0.66 ± 0.12 ^c^ | 1.31 ± 0.28 ^a^ |
| 2-Methyl-1-propanol (mg l^-1^) | 3.6 ± 1.1 ^c^ | 4.1 ± 0.6 ^c^ | 3.6 ± 0.7 ^c^ | 4.6 ± 0.4 ^c^ | 4.6 ± 1.7 ^c^ | 6.8 ± 1.6 ^b^ | 3.8 ± 0.5 ^c^ | 8.8 ± 1.6 ^a^ |
| Isoamyl alcohol (mg l^-1^) | 19.4 ± 6.3 ^cd^ | 22.4 ± 4.6 ^cd^ | 17.8 ± 1.9 ^cd^ | 24.1 ± 0.5 ^bc^ | 22.4 ± 7.5 ^cd^ | 32.5 ± 5.9 ^a^ | 14.5 ± 1.9 ^d^ | 31.4 ± 4.0 ^ab^ |
| Ethyl acetate (mg l^-1^) | 8.4 ± 4.0 ^b^ | 11.2 ± 2.7 ^b^ | 8.4 ± 1.5 ^b^ | 15.1 ± 1.3 ^a^ | 9.9 ± 0.9 ^b^ | 14.8 ± 0.5 ^a^ | 3.5 ± 0.5 ^c^ | 17.0 ± 2.0 ^a^ |
| Isoamyl acetate (mg l^-1^) | 0.073 ± 0.007 ^bcd^ | 0.073 ± 0.005 ^bcd^ | 0.067 ± 0.003 ^cd^ | 0.076 ± 0.004 ^bc^ | 0.073 ± 0.008 ^bcd^ | 0.091 ± 0.013 ^a^ | 0.062 ± 0.002 ^d^ | 0.082 ± 0.008 ^ab^ |
| Ethyl hexanoate (mg l^-1^) | 0.17 ± 0.02 | 0.19 ± 0.02 | 0.15 ± 0.01 | 0.17 ± 0.01 | 0.15 ± 0.02 | 0.16 ± 0.02 | 0.13 ± 0.01 | 0.17 ± 0.02 |
| Ethyl octanoate (mg l^-1^) | 0.45 ± 0.17 ^ab^ | 0.46 ± 0.17 ^ab^ | 0.35 ± 0.10 ^bc^ | 0.49 ± 0.11 ^ab^ | 0.36 ± 0.05 ^bc^ | 0.57 ± 0.03 ^a^ | 0.24 ± 0.04 ^c^ | 0.49 ± 0.09 ^ab^ |
| Glycerol/ethanol (mmol/mol) | 0.063 ± 0.007 | 0.066 ± 0.026 | 0.071 ± 0.004 | 0.060 ± 0.009 | 0.070 ± 0.004 | 0.058 ± 0.005 | 0.081 ± 0.012 | 0.063 ± 0.004 |
| Lactic acid/ethanol (mmol/mol) | 0.107 ± 0.010 ^a^ | 0.085 ± 0.005 ^c^ | 0.113 ± 0.004 ^a^ | 0.093 ± 0.001 ^bc^ | 0.106 ± 0.017 ^ab^ | 0.088 ± 0.002 ^c^ | 0.113 ± 0.004 ^a^ | 0.090 ± 0.003 ^c^ |
| Acetic acid/ethanol (mmol/mol) | 0.090 ± 0.015 ^abc^ | 0.070 ± 0.009 ^d^ | 0.101 ± 0.005 ^a^ | 0.078 ± 0.002 ^bcd^ | 0.094 ± 0.021 ^ab^ | 0.074 ± 0.004 ^cd^ | 0.097 ± 0.008 ^a^ | 0.062 ± 0.006 ^d^ |
| Acetic acid/lactic acid (mmol/mol) | 0.84 ± 0.06 ^a^ | 0.82 ± 0.07 ^a^ | 0.90 ± 0.03 ^a^ | 0.84 ± 0.02 ^a^ | 0.88 ± 0.09 ^a^ | 0.84 ± 0.03 ^a^ | 0.86 ± 0.08 ^a^ | 0.68 ± 0.04 ^b^ |
| D-lactic acid (% of total) | 40.4 ± 1.7 ^e^ | 42.2 ± 1.0 ^de^ | 46.2 ± 1.4 ^ab^ | 44.9 ± 1.0 ^bc^ | 47.4 ± 0.5 ^a^ | 45.9 ± 0.5 ^ab^ | 43.9 ± 1.2 ^cd^ | 46.0 ± 1.1 ^ab^ |
